# *TaPIK3AP* Regulates Female Reproduction in *Tuta absoluta* Through Juvenile Hormone-, Vitellogenin-, and TOR-Related Signaling

**DOI:** 10.3390/insects17070711

**Published:** 2026-07-10

**Authors:** Jing Li, Jiahui Song, Li Yang, Zhuting Zhang, Guy Smagghe, Wenjia Yang

**Affiliations:** 1 College of Life and Health Science, Kaili University, Kaili 556011, China; lijing4821@163.com (J.L.); zhangzhuting120@163.com (Z.Z.); 2 Guizhou Key Laboratory of Agricultural Biosecurity, Key Laboratory of Surveillance and Management of Invasive Alien Species in Guizhou Province, College of Biological and Environmental Engineering, Guiyang University, Guiyang 550005, China; songjiahui199701@126.com; 3 Institute of Entomology, Guizhou University, Guiyang 550025, China; guysma9@gmail.com; 4 Department of Biology, Vrije Universiteit Brussel (VUB), 1050 Brussels, Belgium

**Keywords:** *Tuta absoluta*, *PIK3AP*, RNA interference, PI3K-Akt signaling pathway, juvenile hormone, *vitellogenin*, TOR signaling pathway

## Abstract

*Tuta absoluta* is a globally significant invasive pest that has rapidly developed resistance to multiple classes of insecticides, highlighting the critical need for RNA interference (RNAi) targets for sustainable pest management. The insulin signaling pathway is a key regulator of insect reproduction; however, the role of PIK3AP, an adaptor protein that links receptor tyrosine kinases to the PI3K-Akt signaling pathway, remains poorly understood in Lepidopteran pests. In this study, *TaPIK3AP* was identified in *T. absoluta*, exhibiting elevated expression levels in the heads of female adults and during the early reproductive period. RNAi-mediated knockdown of *TaPIK3AP* reduced fecundity by 76%, decreased egg hatching rate by 43%, shortened the oviposition period by two days, and caused pronounced ovarian atrophy accompanied by impaired yolk deposition. Mechanistically, knockdown of *TaPIK3AP* led to the suppression of *Vg* and *VgR* expression, a reduction in juvenile hormone (JH) titer, downregulation of JH signaling genes, and a significant decrease in the transcript levels of key components of the target of rapamycin (TOR) pathway, including *mTOR*, *Rheb*, and *S6K*. These findings demonstrate that *TaPIK3AP* integrates Vg, JH, and TOR signaling pathways to regulate female reproduction in *T. absoluta*, thereby identifying it as a potential molecular target for RNAi-based sustainable pest management strategies.

## Simple Summary

*Tuta absoluta*, commonly known as the South American tomato leafminer, represents a destructive invasive pest that has developed resistance to numerous conventional insecticides, complicating control efforts and underscoring the necessity for alternative strategies. This study investigated *TaPIK3AP*, a gene involved in the insulin signaling pathway, and its role in female reproductive processes. We found that *TaPIK3AP* is highly active in female adult heads and during the early egg-laying stage. Knocking down this gene through RNA interference resulted in a marked reduction in egg production, decreased egg hatchability, and a shortened oviposition period. Additionally, the ovaries displayed underdevelopment with reduced yolk formation. Further analyses revealed that *TaPIK3AP* influences reproduction through two mechanisms: directly by regulating vitellogenin and its receptor, which are essential for egg development, and indirectly by affecting juvenile hormone levels and the TOR signaling pathway.

## 1. Introduction

*Tuta absoluta* (Meyrick), commonly known as the South American tomato leafminer, is a moth species within the family Gelechiidae. Originally endemic to Peru, it has emerged as one of the most destructive invasive agricultural pests worldwide, with larval infestations capable of causing up to 100% crop loss if not effectively managed. The species has successfully colonized more than 100 countries and regions across Europe, Africa, Western Asia, and both South and Central America [1,2]. Its rapid and widespread invasion is largely due to its pronounced ecological plasticity, which allows it to thrive under diverse climatic conditions, coupled with efficient human-mediated dispersal via trade of infested seedlings, fruits, and other plant materials. This species primarily targets solanaceous crops, with tomato representing its principal and most economically important host [3]. Additionally, it poses a threat to other solanaceous crops such as potato, eggplant, and pepper, thereby amplifying its impact on global agricultural productivity [4]. The larval stage is particularly destructive, as larvae mine the mesophyll tissues of leaves and feed within stems and developing vegetables, producing characteristic serpentine mines accompanied by chlorosis, wilting, and severe skeletonization [5]. Such feeding damage can result in 20–30% yield loss under moderate infestation and exceed 50%, or even lead to total crop failure under severe outbreak conditions [6]. Chemical control remains the dominant management strategy due to its rapid efficacy and operational convenience. However, intensive and prolonged use of broad-spectrum insecticides has driven the rapid evolution of resistance to multiple insecticide classes, primarily through enhanced detoxification enzyme activity and target-site modifications. Moreover, such practices disrupt natural enemy populations and pose significant risks to environmental integrity and human health [7], underscoring the urgent need for environmentally sustainable and molecular targeted pest management strategies.

Identification of key genes involved in insect reproductive processes and the elucidation of their molecular functions have enabled the discovery of novel insecticidal targets, and provided innovative strategies for pest control [8,9]. Among these targets, insulin signaling emerges as a highly conserved regulatory system. Insulin, a multifunctional peptide hormone, interacts with membrane-bound receptors to activate downstream signaling cascades that regulate growth, metabolism, development, reproduction, and stress responses in insects [10,11]. Two principal downstream pathways, the mitogen-activated protein kinase (MAPK) and phosphoinositide 3-kinase (PI3K) pathways, mediate insulin signal transduction. Although these pathways can function independently, extensive crosstalk exists between them. Notably, the activation of MAPK is dependent on PI3K signaling activity [12], indicating that PI3K functions as a central axis in the insect insulin signaling pathway.

The PI3K-Akt pathway, as a core component of insulin signaling, functions as a key regulatory hub in insect reproductive development [13,14]. Beyond its interaction with juvenile hormone (JH) and ecdysteroids, this pathway integrates nutritional and endocrine cues to orchestrate reproductive processes. It exerts direct regulatory effects on reproduction and operates synergistically with JH and ecdysteroid signaling. In *Nilaparvata lugens*, the transcription factor Forkhead Box O (FoxO), a downstream effector of PI3K-Akt signaling, binds to the exon region of the *vitellogenin* (*Vg*) gene, inhibiting its transcription and consequently reducing oviposition and egg hatching success [15]. Similar mechanisms have been identified in *Tribolium castaneum* [16], indicating a conserved role for PI3K-Akt signaling in insect reproductive regulation, independent of traditional endocrine pathways. Furthermore, the PI3K-Akt signaling cascade interacts with the target of rapamycin (TOR) pathway, ecdysteroid signaling, and JH signaling, particularly during vitellogenesis and oocyte maturation [17]. Previous studies have established that the insulin and amino acid/TOR (AA/TOR) signaling pathways are critical for Vg synthesis and oocyte development in insects [18]. In *Blattella germanica*, RNA interference (RNAi)-induced silencing of *FoxO* results in elevated JH biosynthesis and a marked upregulation of *Vg* expression [19]. Similarly, in *Leptinotarsa decemlineata*, FoxO mediates the larva-pupa-adult transition. Its silencing represses 20-hydroxyecdysone (20E) signaling and leads to developmental arrest, indicating its pivotal role in metamorphosis [20].

As an important node connecting receptor tyrosine kinases with the PI3K-Akt and MAPK signaling cascades, the *PIK3AP* gene is implicated in insect development, growth regulation, and potentially in reproductive control [21]. Structurally, PIK3AP is characterized by a conserved DBB (Dof, BCAP, and BANK) domain, named after its homologs in the *Drosophila* Dof and vertebrate BCAP and BANK proteins [22]. The Dof protein plays an essential role in insect development; for instance, in *Drosophila melanogaster*, its phosphotyrosine motifs are essential for embryogenesis via fibroblast growth factor receptor (FGFR)-dependent MAPK activation [21,23]. Despite the conserved role of *PIK3AP* in signaling, its specific function in insect reproductive regulation remains poorly understood, particularly in non-model Lepidopteran pests. Although PI3K-Akt signaling is widely recognized for its involvement in various physiological processes, including reproduction across eukaryotes, the role of *PIK3AP* in insects, especially within Lepidopteran species, has been scarcely investigated. Consequently, its regulatory contribution to reproductive development remains uncharacterized. In this study, we identified the *TaPIK3AP* gene from *T. absoluta* and analyzed its spatial and temporal expression patterns. Furthermore, RNAi was employed to assess its functional roles in female reproductive traits, including ovarian development, fecundity, and egg hatchability. This study aims to investigate the molecular regulatory mechanisms of *TaPIK3AP* in the reproductive processes of *T. absoluta* and to evaluate its potential as a candidate target for RNAi-based pest management strategies against this highly destructive invasive species.

## 2. Materials and Methods

### 2.1. Insects

The *T. absoluta* population was originally collected from Kunming City, Yunnan Province, China, and has been continuously maintained under greenhouse conditions since establishment. Larvae were reared on tomato leaves, while adults were supplied with 10% (*w*/*v*) honey solution. Rearing conditions were maintained at 26 ± 1 °C, 60 ± 5% relative humidity, and a photoperiod of 16 h light followed by 8 h darkness [24].

### 2.2. Cloning and Bioinformatics Analysis of TaPIK3AP

Taking the amino acid sequence of *D*. *melanogaster* PIK3AP as a query, the TBLASTN program in TBtools v2.23+ was used to search the assembled *T. absoluta* transcriptome database to identify candidate homologous sequences with high similarity. Candidate homologous sequences were verified by reciprocal BLAST against the NCBI BLAST platform (https://blast.ncbi.nlm.nih.gov/, accessed on 7 June 2026). Candidate open reading frames (ORFs) of *TaPIK3AP* were amplified using gene-specific primers (Table S1) by polymerase chain reaction (PCR).

PCR was carried out in a total volume of 50 µL, consisting of 25.0 µL of 2× EasyTaq^®^ PCR SuperMix (+dye) (TransGen Biotech, Beijing, China), 21.0 µL of ddH_2_O, 2.0 µL of cDNA template, and 1.0 µL of each of forward and reverse primers (10 µM). The thermal cycling program included an initial denaturation step at 94 °C for 3 m, followed by 35 cycles of denaturation at 95 °C for 30 s, annealing at 55 °C for 30 s, and extension at 72 °C for 2 m, with a final extension at 72 °C for 10 m. The resulting PCR products were separated by agarose gel electrophoresis, inserted into the pGEM-T Easy vector (Promega, Madison, WI, USA), and introduced into *Escherichia coli* Trans5α competent cells (TransGen Biotech, Beijing, China). Positive single colonies were selected and grown, and the plasmids were extracted and submitted to Tsingke Biotechnology Co., Ltd. (Beijing, China) for sequencing.

Conserved domains of TaPIK3AP were predicted using the SMART database (http://smart.embl-heidelberg.de/, accessed on 9 June 2026). The physical and chemical properties of the TaPIK3AP protein were predicted using the Expasy ProtParam platform (https://web.expasy.org/protparam/, accessed on 9 June 2026). The DBB domain sequences of PIK3AP from representative species of Lepidoptera, Coleoptera, Diptera, Hymenoptera, and Hemiptera were aligned using CLC Genomics Workbench 12. Multiple sequence alignment was performed using ClustalW in MEGA 11, after which a phylogenetic tree was constructed using the neighbor-joining method and branch support was assessed using 1000 bootstrap replicates [25].

### 2.3. Spatio-Temporal Expression Pattern

Samples representing various developmental stages of *T. absoluta*, including entire insects of 1st–4th instar larvae, 1–7-day-old female pupae, and 1–8-day-old female adults, were systematically collected. Each developmental stage sample comprised 15–50 individuals, with three biological replicates per stage. For tissue-specific expression analysis, samples of 2-day-old female adults were meticulously dissected under a stereomicroscope, including head, gut, Malpighian tubules, fat body, abdomen, wings, ovaries, and epidermis. Three biological replicates were prepared. Total RNA was extracted using TransZol Up reagent (TransGen Biotech, Beijing, China). The concentration and purity of RNA were evaluated using a NanoDrop 2000c spectrophotometer (Thermo Fisher Scientific, Wilmington, MA, USA), and RNA integrity was verified by 1% agarose gel electrophoresis. First-strand cDNA was synthesized using the TransScript^®^ One-Step gDNA Removal and cDNA Synthesis SuperMix Kit (TransGen Biotech). Quantitative real-time PCR (qPCR) was conducted to assess the expression levels of *TaPIK3AP* gene. The qPCR reaction mixture comprised 2 μL of cDNA template, 10 μL of TransStart^®^ Top Green qPCR SuperMix (TransGen Biotech), 2 μL each of forward and reverse primers (Table S1), and 6 μL of RNase-free water. The thermal cycling protocol included an initial denaturation at 95 °C for 5 min, followed by 40 cycles of 95 °C for 10 s and 60 °C for 30 s. To verify amplification specificity, a melting curve analysis was performed from 65 °C to 95 °C. The *TaEF1α* gene (*Elongation factor 1 alpha*; GenBank accession no. MZ054826) served as the internal reference gene [26]. The reactions were executed using the CFX96^TM^ Real-Time PCR Detection System (Bio-Rad, Hercules, CA, USA). Relative expression levels were calculated using the 2^−ΔΔCt^ method [27].

### 2.4. RNA Interference

Double-stranded RNA (dsRNA) targeting *TaPIK3AP* was synthesized using the TranscriptAid T7 High Yield Transcription Kit (Thermo Fisher Scientific, Wilmington, DE, USA). The dsRNA was purified via phenol–chloroform extraction. For RNAi assays, dsRNA was microinjected into 2-day-old female pupae using a Nanoliter 2010 microinjector (World Precision Instruments, Sarasota, FL, USA), with each pupa receiving 500 ng of dsRNA. An equivalent amount of ds*GFP* (*green fluorescent protein*) was injected as a negative control. Each experimental group comprised 30 pupae, with three biological replicates conducted. Post-injection, the pupae were transferred to rearing containers containing sterile soil. The soil surface was lightly misted with sterile water to maintain humidity, and the containers were placed in an artificial climate chamber under controlled conditions to facilitate subsequent development.

### 2.5. Phenotypic Observation of T. absoluta

At 48 h post-injection of ds*TaPIK3AP*, 20 pupae were randomly collected and immediately frozen in liquid nitrogen for rapid preservation. Total RNA was extracted and reverse-transcribed, and the expression level of *TaPIK3AP* was quantified by qPCR to evaluate RNAi silencing efficiency. After adult emergence, newly eclosed females were paired with age-matched males at a ratio of 1:3 (female: male), with 30 pairs set up for each treatment group, and maintained under the same rearing conditions. Once the adults began oviposition, fecundity parameters were recorded every 24 h, including total number of eggs laid per female throughout her lifespan, daily egg production, oviposition duration, and egg hatching rate. For each treatment group, three biological replicates were performed. In addition, female adults at 2 days post-emergence were dissected. Ovarian morphology was examined, and the lengths of ovarian tubules and eggs were measured using a VHX-6000 digital microscope system (Keyence, Osaka, Japan). Ovarian development stages were classified following the criteria [28]. Representative images of ovarian structures were captured for morphological analysis.

### 2.6. Determination of Vg Content

ds*TaPIK3AP* was injected into female pupae, with ds*GFP* injected as a negative control. On the first day after adult emergence, 0.05 g of female adults (25–30 adults) from both control and RNAi treatment groups were collected. Vg content was quantified using an insect vitellogenin enzyme-linked immunosorbent assay (ELISA) kit (Shanghai Enzyme-linked Biotechnology Co., Ltd., Shanghai, China), according to the manufacturer’s instructions. Briefly, each sample was homogenized in phosphate-buffered saline (PBS) and centrifuged, and the supernatant was used for ELISA analysis. Absorbance was measured at 450 nm using a SpectraMax L microplate reader (Molecular Devices, Sunnyvale, CA, USA). Three biological replicates were performed for each treatment.

### 2.7. Determination of Total JH Titer

At 48 h post-injection of ds*TaPIK3AP*, 0.05 g of female pupae (30–35 pupae) from both ds*GFP* control and RNAi treatment groups were collected, with three biological replicates. For total JH titer determination, 500 μL of 1× PBS buffer was added to each sample, followed by homogenization using a high-speed, low-temperature tissue grinder. JH titer was quantified using an Insect Juvenile Hormone ELISA Kit (Shanghai Enzyme-linked Biotechnology, China), according to the manufacturer’s instructions. Absorbance was measured at 450 nm using a microplate reader.

### 2.8. Expression Levels of Vg, VgR, TOR, and JH Signaling Genes

Female pupae were collected at 48 h post-injection of ds*TaPIK3AP*. After total RNA extraction and reverse transcription, qPCR was performed to examine the expression levels of target genes. These genes were related to the *vitellogenin* (*Vg*), *vitellogenin receptor* (*VgR*), and genes associated with the JH signaling pathway, namely *juvenile hormone acid methyltransferase* (*JHAMT*) and the *transcription factor Krüppel homolog 1* (*Kr-h1*). Additionally, the TOR signaling pathway genes, specifically *mechanistic target of rapamycin* (*mTOR*), *regulatory-associated protein of mTOR* (*RAPTOR*), *Ras homolog enriched in brain* (*Rheb*), *tuberous sclerosis complex 1* (*TSC1*), *tuberous sclerosis complex 2* (*TSC2*), *AMP-activated protein kinase* (*AMPK*), *ribosomal protein S6 kinase* (*S6K*), *eukaryotic translation initiation factor 4E-binding protein* (*4EBP*), and *serine/threonine-protein kinase* (*AKT*), were also detected.

### 2.9. Data Analysis

Statistical analyses were performed using SPSS version 25.0 (IBM, Armonk, NY, USA). Expression levels of *TaPIK3AP* across developmental stages and tissues were analyzed using one-way analysis of variance (ANOVA) followed by Tukey’s honestly significant difference (HSD) post hoc test. Egg hatching rates between the two groups were analyzed using the Mann–Whitney U test. For all other comparisons between the ds*TaPIK3AP* and ds*GFP* control groups (including number of eggs, oviposition period, JH titer, Vg content, lengths of ovarian tubules and eggs, and expression levels of *Vg*, *VgR*, JH signaling genes, and TOR pathway genes), Student’s *t*-test was applied (* *p* < 0.05; ** *p* < 0.01; *** *p* < 0.001).

## 3. Results

### 3.1. Sequence and Phylogenetic Analysis of TaPIK3AP

**Figure 1:**
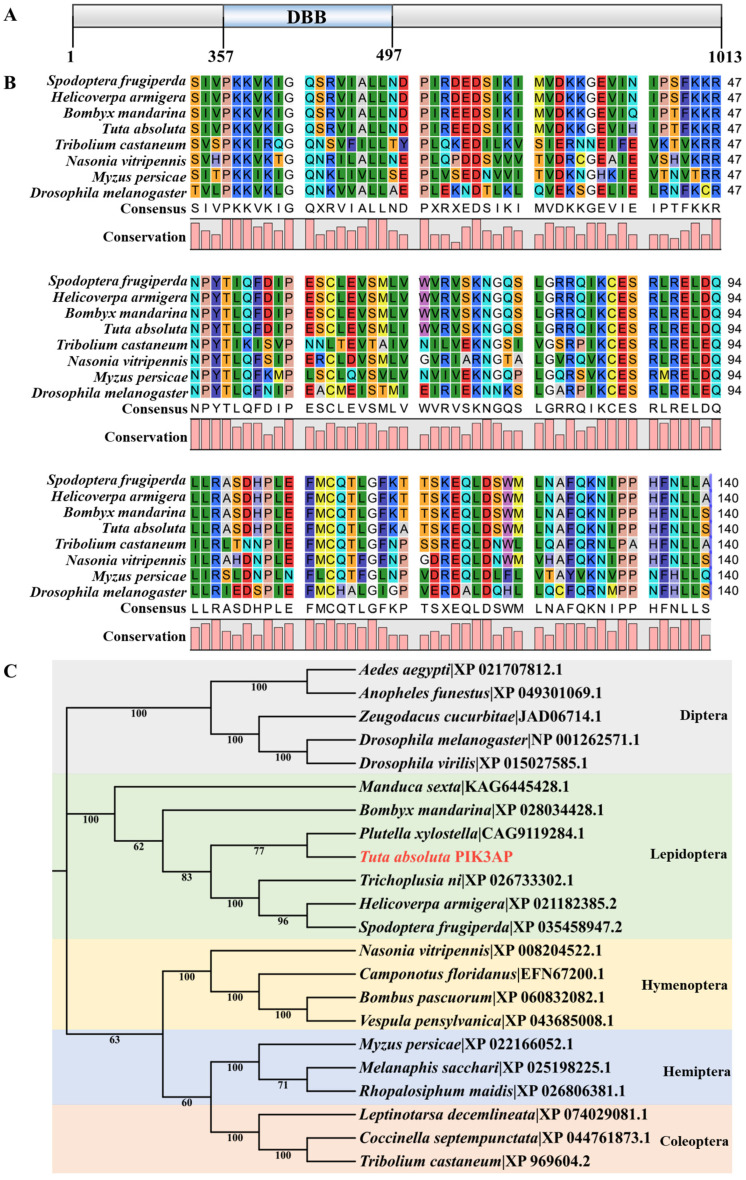
Protein sequence and phylogenetic analysis of TaPIK3AP from *Tuta absoluta*. (**A**) Schematic representation of the conserved domains of the TaPIK3AP protein, including DBB (Dof, BCAP, and BANK) domain. (**B**) Alignment protein sequence analysis and DBB domain of PIK3AP. (**C**) Phylogenetic analysis of PIK3AP proteins from representative insect species across multiple orders. The phylogenetic tree was constructed using the neighbor-joining method with 1000 bootstrap replicates. GenBank accession number of each species is listed in the tree. Red represents the PIK3AP of *T. absoluta*.

The open reading frame of the *TaPIK3AP* gene comprises 3042 bp, which encodes a protein of 1013 amino acid residues, with a predicted molecular mass of 115.23 kDa and a theoretical isoelectric point of 6.60. Bioinformatic analysis revealed the presence of DBB domain located at amino acid positions 357–497 (Figure 1A). The DBB domain of TaPIK3AP in *T. absoluta* was compared at the amino acid level with homologous sequences from representative species of Lepidoptera, Coleoptera, Diptera, Hymenoptera, and Hemiptera. The results showed that the DBB domain of *T. absoluta* TaPIK3AP is highly conserved and exhibits strong sequence similarity with orthologs from other insect species, containing multiple conserved amino acid regions, indicating a high level of evolutionary conservation (Figure 1B). A phylogenetic tree constructed using PIK3AP amino acid sequences from the same insect orders demonstrated that sequences clustered according to taxonomic grouping. Notably, *T. absoluta* TaPIK3AP clustered with Lepidopteran orthologs and showed the closest phylogenetic relationship to PIK3AP from *Plutella xylostella* (Figure 1C).

### 3.2. Spatiotemporal Expression Analysis of TaPIK3AP

**Figure 2:**
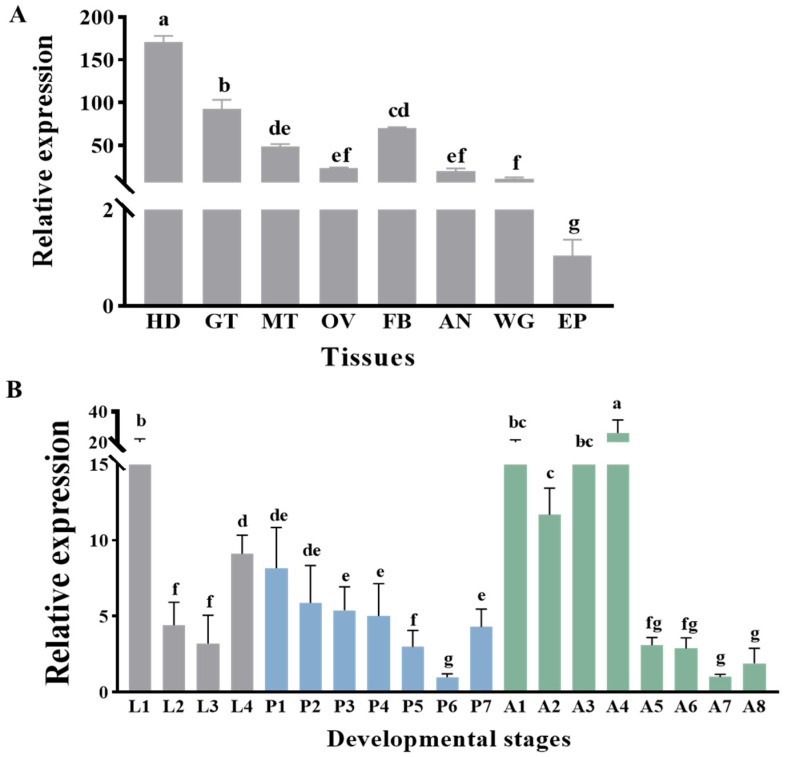
Expression patterns of *TaPIK3AP* across different tissues and developmental stages of *T. absoluta*. (**A**) Relative expression levels of *TaPIK3AP* in different tissues of 2-day-old female adults. HD: head. GT: gut. MT: Malpighian tubules. OV: ovary. FB: fat body. AN: abdomen. WG: wing. EP: epidermis. (**B**) Relative expression levels of *TaPIK3AP* at different developmental stages, for which whole bodies (entire insects) were used as samples. L1–4: first- to fourth-instar larvae. P1–7: 1–7-day-old pupae. A1–8: 1–8-day-old female adults. Data are presented as mean ± SE. Different letters indicate significant differences among samples (*p* < 0.05).

The results indicated that *TaPIK3AP* was expressed in all tested tissues, with the highest expression detected in the head, followed by the gut, fat body, and Malpighian tubules, while the lowest expression level was observed in the epidermis (Figure 2A). Regarding developmental expression patterns, *TaPIK3AP* transcripts were detected at all examined stages. Overall, expression showed a trend of initial decrease followed by an increase across larval, pupal, and adult stages. Specifically, relatively high expression levels were observed in first-instar larvae and in 1-, 3-, and 4-day-old adults, whereas markedly reduced expression was detected in 6-day-old pupae, 7-day-old adults, and 8-day-old adults (Figure 2B).

### 3.3. The Effect of dsRNA-Mediated Knockdown of TaPIK3AP on the Reproduction of T. absoluta

**Figure 3:**
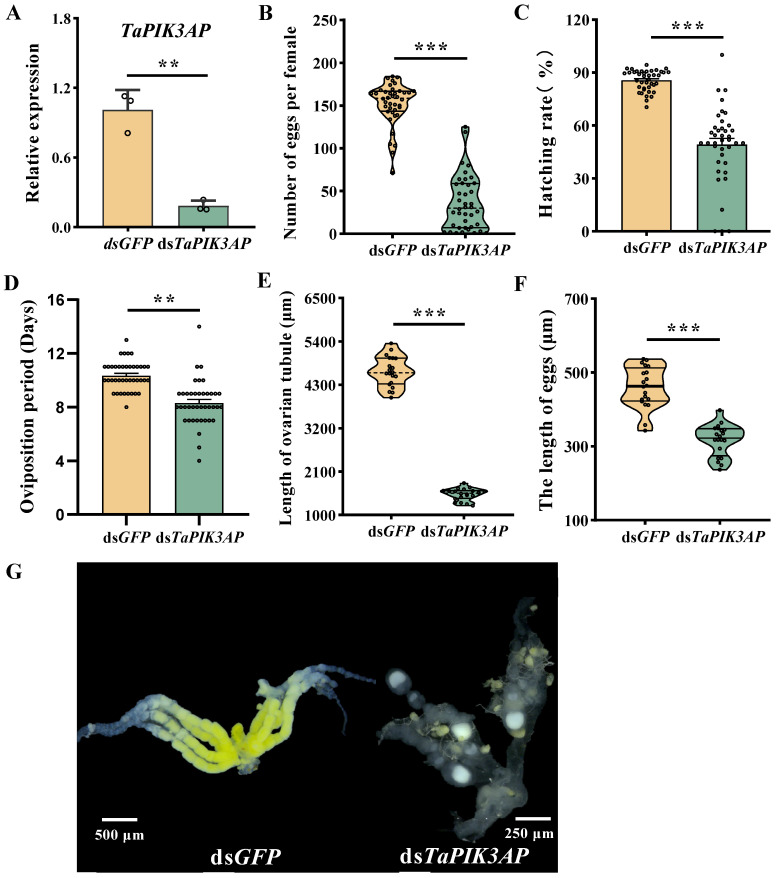
Effects of *TaPIK3AP* knockdown on female fecundity and ovarian development of *T. absoluta*. (**A**) RNAi silencing efficiency of *TaPIK3AP* at 48 h. (**B**) Number of eggs laid per female. (**C**) Egg hatching rate. (**D**) Oviposition period. (**E**) The lengths of ovarian tubules. (**F**) The lengths of eggs. (**G**) Morphological observations of ovarian development. Data are presented as mean ± SE. Data points represent the biological replicates. Asterisks indicate significant differences (** *p* < 0.01, *** *p* < 0.001).

To investigate the function of *TaPIK3AP* in the reproductive process of *T. absoluta* females, ds*TaPIK3AP* was injected into female pupae. Compared with the ds*GFP* control group, *TaPIK3AP* transcript levels were reduced by 82% at 48 h post-injection (Figure 3A). Knockdown of *TaPIK3AP* resulted in a significant 76% reduction in female fecundity (number of eggs per female, Figure 3B), a 43% decrease in egg hatching rate (Figure 3C), a reduction in the oviposition period by 2 days (Figure 3D), and a decrease in both ovarian tubule and egg length (Figure 3E,F). Suppression of *TaPIK3AP* led to notable shrinkage of ovaries, accompanied by the degeneration of fat bodies. Ovaries in the RNAi group predominantly remained at stage II, characterized by whitish immature oocytes and few developed eggs. In contrast, control group ovaries exhibited advanced development, with enlarged ovarioles, abundant yolk deposition, and progression to stage III (oviposition peak), containing numerous mature oocytes (Figure 3G).

### 3.4. Effect of dsRNA-Mediated Knockdown of TaPIK3AP on Vg, VgR, JH and TOR Pathway of T. absoluta

**Figure 4:**
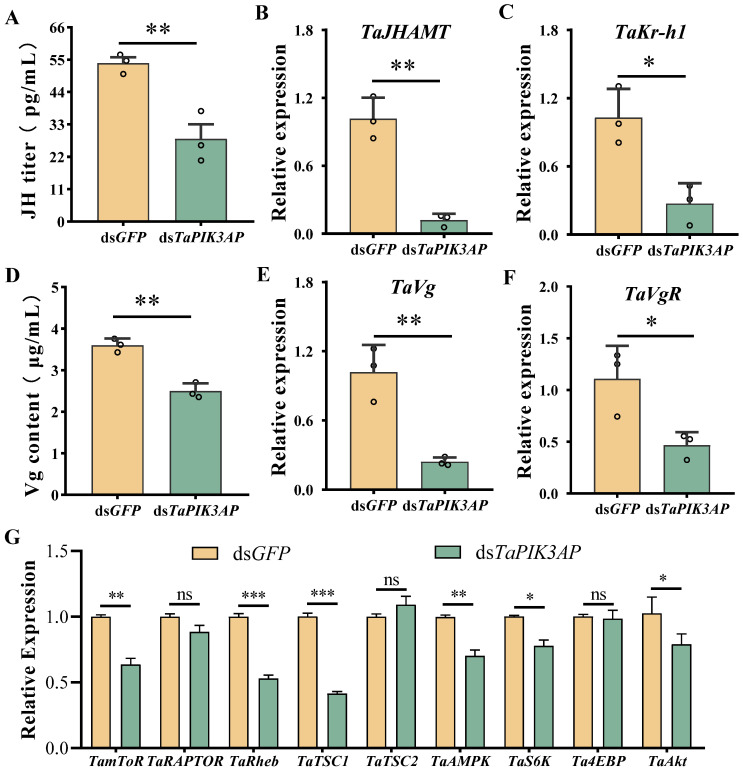
Effects of *TaPIK3AP* knockdown on JH, Vg, and TOR signaling pathway. (**A**) JH titer. (**B**) Relative expression levels of *TaJHAMT*. (**C**) Relative expression levels of *TaKr-h1*. (**D**) Vg content. (**E**) Relative expression levels of *Vg*. (**F**) Relative expression levels of *VgR*. (**G**) Relative expression levels of nine genes involved in the TOR signaling pathway. The transcript levels of the above genes in ds*GFP*-treated pupae were normalized to 1, as indicated by the red dotted line. Data are presented as mean ± SE. Data points represent the biological replicates. Asterisks indicate significant differences (ns, not significant; * *p* < 0.05, ** *p* < 0.01, *** *p* < 0.001).

Upon knockdown of *TaPIK3AP*, the total JH content in female pupae at 48 h post-injection of *T. absoluta* was significantly reduced by 48% compared to the ds*GFP* group (Figure 4A). Concurrently, the transcript levels of JH signaling genes, *TaJHAMT* and *TaKr-h1*, in the same female pupae were markedly downregulated by 88% and 73%, respectively (Figure 4B,C). Additionally, Vg content in 1-day-old female adults decreased by 31% (Figure 4D), with the expression levels of *Vg* and *VgR* reduced by 76% and 53%, respectively (Figure 4E,F). Furthermore, at 48 h post-injection of female pupae, there was a significant transcriptional downregulation of *TaToR*, *TaRheb*, *TaTSC1*, *TaAMPK*, *TaS6K*, and *TaAkt*, while no significant changes were observed in the expression levels of *TaRAPTOR*, *TaTSC2*, or *Ta4EBP* (Figure 4G).

## 4. Discussion

In the present study, *PIK3AP* was identified from the transcriptome of *T. absoluta*, showing high conservation across representative insect orders, including Coleoptera, Hemiptera, Hymenoptera, Diptera, and Lepidoptera. In particular, the amino acid sequence of the DBB domain exhibited strong similarity among species and was homologous to the *Drosophila* Dof protein (Downstream of FGFR) [21,22]. This conserved domain likely confers TaPIK3AP the capacity to fine-tune PI3K activation during reproductive development, thereby linking nutritional cues to energy allocation for Vg synthesis and ovarian maturation. Phylogenetic analysis further indicated that TaPIK3AP clusters closely with Lepidopteran homologs, suggesting conserved evolutionary origin and potentially similar biological functions within this order. Consistent with this hypothesis, *TaPIK3AP* showed elevated expression in 1–4-day-old female adults, corresponding to the active ovarian development stage and peak oviposition period. Similar expression patterns of PI3K/Akt/TOR pathway components have been reported in *Hyphantria cunea* (*HcPI3K*, *HcAKT*, and *HcFoxO*) [29], *Sogatella furcifera* (*SfAkt*) [30], *Frankliniella occidentalis* (*FoAKT* and *FoPDK*) [31], and *Cyrtorhinus lividipennis* (*ClInR*, *ClPI3K*, and *ClAKT*) [32], where high expression during adulthood supports reproductive processes.

Tissue-specific expression analysis revealed that *TaPIK3AP* is broadly expressed in eight tissues, suggesting functional pleiotropy. Notably, the highest expression was detected in the head, which may be associated with the central role of the brain in insulin-like peptide production and neuroendocrine signaling [33,34]. Similar head-biased expression has been reported for *B. mori* Akt, which is implicated in reproductive regulation. In contrast, *D. melanogaster* dof [35] and PI3K/Akt pathway genes in *H. cunea* [21] show predominant expression in the gut, indicating potential species-specific divergence in functional specialization, possibly extending beyond reproduction to immunity and metabolic regulation.

The insulin signaling pathway is a central regulator of insect reproduction [36]. Functional evidence from multiple insects supports its conserved role in ovarian development and fecundity. For example, RNAi-mediated silencing of insulin pathway gene *LsAkt* in *Lasioderma serricorne* leads to ovarian atrophy and reduced fecundity [37], while silencing *BIGFLP* in *B. mori* reduces ovarian mass and oviposition [38]. Similarly, suppression of *HcPI3K*, *HcAkt*, and *HcFoxO* in *H. cunea* significantly decreases reproductive output [29]. In the present study, suppressing the expression of *TaPIK3AP* resulted in reduced fecundity, decreased egg hatchability, and shortened oviposition duration in *T. absoluta*. Morphological observations further revealed reduced ovariolar length, smaller eggs, and abnormal ovarian structure, confirming that disruption of *TaPIK3AP* impairs ovarian development and reproductive capacity. These findings strongly support the hypothesis that *TaPIK3AP* functions as an upstream regulator of reproductive signaling within the insulin pathway.

Beyond direct regulation of oogenesis, insulin signaling interacts extensively with vitellogenesis through modulation of *Vg* and *VgR* expression. In *Maruca testulalis*, suppression of *MtInR*, *MtAkt*, *MtFoxO*, and *MtTOR* leads to reduced Vg/VgR expression and impaired ovarian development [39]. Similarly, in *Coridius chinensis*, silencing *CcAkt* decreases *Vg*/*VgR* expression and stunted the development of the ovarioles [40]. Consistently, knockdown of *TaPIK3AP* significantly reduced *Vg* and *VgR* transcript levels in *T. absoluta*, accompanied by decreased Vg content and impaired ovarian development. These results suggest that *TaPIK3AP* regulates reproduction at least partly through modulation of vitellogenesis.

In addition to this direct regulatory axis, insulin signaling coordinates reproduction with TOR, JH, and ecdysteroid pathways. The TOR pathway is a well-established nutrient sensor controlling insect reproduction [41]. In *Aedes aegypti*, silencing TOR pathway genes (*TOR*, *Rheb*, and *S6K*) suppresses *Vg* expression and reduces fecundity [42,43]. In *N. lugens*, TOR signaling also promotes JH biosynthesis, linking nutrition to reproductive output [44]. In *D. melanogaster*, silencing *InR1* in the corpus allatum modulates JH-hydrolyzing activity in response to heat stress and reduces female fecundity; however, JH application restores fecundity in these females [45].

In the present study, reducing the expression of *TaPIK3AP* led to significant transcriptional downregulation of TOR pathway genes, accompanied by reduced expression of JH biosynthesis and signaling genes *TaJHAMT* and *TaKr-h1*, decreased total JH titer, and suppression of vitellogenesis. These findings indicate that *TaPIK3AP* not only directly regulates reproductive processes but also indirectly modulates JH-mediated endocrine signaling. Comparable regulatory interactions have been reported in *Spodoptera litura*, where *SlInR* silencing reduces both JH titers and Vg expression [46], and in *L. decemlineata*, where *LdILP2* influences JH signaling and reproductive output [47]. Together, these studies support a conserved model in which PI3K-Akt pathway components integrate nutritional and hormonal signals to regulate insect reproduction through both direct and indirect mechanisms.

Despite these advancements, several limitations persist. Firstly, the upstream regulatory mechanisms governing *TaPIK3AP* expression remain unclear, and its potential interactions with other signaling networks, particularly those mediated by insulin receptors, necessitate further investigation. Secondly, although this study provides compelling evidence for the downstream effects on the TOR and JH pathways, the specific molecular intermediates connecting *TaPIK3AP* to these pathways remain unresolved. Thirdly, the majority of functional evidence is derived from RNAi experiments; thus, employing complementary methodologies such as rescue assays or overexpression systems would enhance causal inference. Although previous studies have confirmed that RNAi effects initiated in early developmental stages can continue into adulthood [48], we did not assess the silencing efficiency at the adult stage, despite observing phenotypic differences. From an applied perspective, although dsRNA injection proves effective under laboratory conditions, its direct application in field settings is hindered by issues related to stability, delivery efficiency, and environmental degradation [49]. Consequently, future research should focus on scalable RNAi delivery strategies, such as spray-induced gene silencing or plant-mediated RNAi systems [50], to assess the practical viability of *TaPIK3AP* as a RNAi target for field-level pest management.

## 5. Conclusions

*TaPIK3AP* is an evolutionarily conserved gene across various insect orders and exhibits distinct spatiotemporal expression patterns in *T. absoluta*, with a strong correlation to reproductive stages. Functional analyses have demonstrated that knocking down *TaPIK3AP* results in the suppression of *Vg* and *VgR* expression, a reduction in JH synthesis, and the transcriptional downregulation of key genes within the TOR pathway. These molecular alterations lead to impaired ovarian development, reduced fecundity, and decreased egg hatchability. Collectively, these findings suggest that knockdown of *TaPIK3AP* is linked to changes in JH-, Vg-, and TOR-related signaling. This may occur through the integration of nutritional and endocrine signaling pathways, ultimately influencing female reproduction in *T. absoluta*. Consequently, *TaPIK3AP* presents as a promising RNAi target for the development of environmentally sustainable and species-specific pest management strategies against *T. absoluta*.

## Data Availability

Data is contained within the article and Supplementary Materials.

## Supplementary Materials

The following supporting information can be downloaded at https://www.mdpi.com/article/10.3390/insects17070711/s1: Table S1. Primer sequences used in this study.

## References

[1] Santana P.A., Kumar L., Da Silva R.S., Picanco M.C. (2019). Global geographic distribution of *Tuta absoluta* as affected by climate change. J. Pest Sci..

[2] Zhang G.F., Ma D.Y., Wang Y.S., Gao Y.H., Liu W.X., Zhang R., Fu W.J., Xian X.Q., Wang J., Kuang M. (2020). First report of the South American tomato leafminer, *Tuta absoluta* (Meyrick), in China. J. Integr. Agric..

[3] Biondi A., Guedes R.N.C., Wan F.H., Desneux N. (2018). Ecology, worldwide spread, and management of the invasive South American tomato pinworm, *Tuta absoluta*: Past, present, and future. Annu. Rev. Entomol..

[4] Desneux N., Wajnberg E., Wyckhuys K.A.G., Burgio G., Arpaia S., Narváez-Vasquez C.A., González-Cabrera J., Ruescas D.C., Tabone E., Frandon J. (2010). Biological invasion of European tomato crops by *Tuta absoluta*: Ecology, geographic expansion and prospects for biological control. J. Pest Sci..

[5] Vivekanandhan P., Swathy K., Sarayut P., Patcharin K. (2024). Biology, classification, and entomopathogen-based management and their mode of action on *Tuta absoluta* (Meyrick) in Asia. Front. Microbiol..

[6] Mansour R., Brévault T., Chailleux A., Cherif A., Grissa-Lebdi K., Haddi K., Mohamed S.A., Nofemela R.S., Oke A., Sylla S. (2018). Occurrence, biology, natural enemies and management of *Tuta absoluta* in Africa. Entomol. Gen..

[7] Phan N.T., Biddinger D.J., Rajotte E.G., Smagghe G., Reddy G.V.P., Ren Z.X. (2025). Pesticide use in integrated pest and pollinator management framework to protect pollinator health. Pest Manag. Sci..

[8] Willow J., Taning C.N.T., Cook S.M., Sulg S., Silva A.I., Smagghe G., Veromann E. (2021). RNAi targets in agricultural pest insects: Advancements, knowledge gaps, and IPM. Front. Agron..

[9] Saini A., Sharma N. (2025). Parental RNA interference (pRNAi) in insects: Mechanisms, applications, and challenges in pest management. Mol. Biol. Rep..

[10] Leyria J., Benrabaa S., Nouzova M., Noriega F.G., Tose L.V., Fernandez-Lima F., Orchard I., Lange A.B., Lange A. (2023). Crosstalk between nutrition, insulin, JH, and ecdysteroid signaling in the classical insect model, *Rhodnius prolixus*. Int. J. Mol. Sci..

[11] Liu F.F., Yu S.X., Chen N., Ren C.H., Li S. (2023). Nutrition- and hormone-controlled developmental plasticity in *Blattodea*. Curr. Opin. Insect Sci..

[12] Badisco L., Van Wielendaele P., Vanden Broeck J. (2013). Eat to reproduce: A key role for the insulin signaling pathway in adult insects. Front. Physiol..

[13] Arik A.J., Hun L.V., Quicke K., Piatt M., Ziegler R., Scaraffia P.Y., Badgandi H., Riehle M.A. (2015). Increased akt signaling in the mosquito fat body increases adult survivorship. FASEB J..

[14] Scieuzo C., Nardiello M., Salvia R., Pezzi M., Chicca M., Leis M., Bufo S.A., Vinson S.B., Rao A., Vogel H. (2018). Ecdysteroidogenesis and development in *Heliothis virescens* (Lepidoptera: Noctuidae): Focus on PTTH-stimulated pathways. J. Insect Physiol..

[15] Dong Y., Chen W.W., Kang K., Pang R., Dong Y.P., Liu K., Zhang W.Q. (2021). FoxO directly regulates the expression of TOR/S6K and *vitellogenin* to modulate the fecundity of the brown planthopper. Sci. China Life Sci..

[16] Sheng Z.T., Xu J.J., Bai H., Zhu F., Palli S.R. (2011). JH regulates vitellogenin gene expression through insulin-like peptide signaling pathway in the red flour beetle, *Tribolium castaneum*. J. Biol. Chem..

[17] Gu S.H., Yeh W.L., Young S.C., Lin P.L., Li S. (2012). TOR signaling is involved in PTTH-stimulated ecdysteroidogenesis by prothoracic glands in the silkworm, *Bombyx mori*. Insect Biochem. Mol. Biol..

[18] Smykal V., Raikhel A.S. (2015). Nutritional control of insect reproduction. Curr. Opin. Insect Sci..

[19] Süren-Castillo S., Abrisqueta M., Maestro J.L. (2012). FoxO inhibits JH biosynthesis and vitellogenin production in the German cockroach. Insect Biochem. Mol. Biol..

[20] Kang W.N., Wang B.Y., Fu K.Y., Guo W.C., Jin L., Li G.Q. (2021). The Leptinotarsa forkhead transcription factor O exerts a key function during larval-pupal-adult transition. J. Insect Physiol..

[21] Csiszar A., Vogelsang E., Beug H., Leptin M. (2010). A novel conserved phosphotyrosine motif in the *Drosophila* fibroblast growth factor signaling adaptor Dof with a redundant role in signal transmission. Mol. Cell. Biol..

[22] Song S.W., Chew C., Dale B.M., Traum D., Peacock J., Yamazaki T., Clynes R., Kurosaki T., Greenberg S. (2011). A requirement for the p85 PI3K adapter protein BCAP in the protection of macrophages from apoptosis induced by endoplasmic reticulum stress. J. Immunol..

[23] Vincent S., Wilson R., Coelho C., Affolter M., Leptin M. (1988). The *Drosophila* protein Dof is specifically required for FGF signaling. Mol. Cell.

[24] Sarmah N., Kaldis A., Taning C.N.T., Perdikis D., Smagghe G., Voloudakis A. (2021). DsRNA-mediated pest management of *Tuta absoluta* is compatible with its biological control agent *Nesidiocoris tenuis*. Insects.

[25] Tamura K., Stecher G., Kumar S. (2021). MEGA11 molecular evolutionary genetics analysis version 11. Mol. Biol. Evol..

[26] Yan X., Zhang Y.B., Xu K.K., Wang Y.W., Yang W.J. (2021). Selection and validation of reference genes for gene expression analysis in *Tuta absoluta* Meyrick (Lepidoptera: Gelechiidae). Insects.

[27] Hellemans J., Mortier G., De Paepe A., Speleman F., Vandesompele J. (2007). qBase relative quantification framework and software for management and automated analysis of real-time quantitative PCR data. Genome Biol..

[28] Yang W.J., Yan X., Han P., Wang M.H., Zhang C., Song J.H., Zhang G.F., Zhang Y.B., Wan F.H. (2024). Ovarian development and role of vitellogenin gene in reproduction of the tomato leaf miner *Tuta absoluta*. Entomol. Gen..

[29] Yan L.Q., Du H., Li Y., Li X., Sun L.L., Cao C.W. (2023). Identification and characterization of key genes in insulin signaling pathway as molecular targets for controlling the fall webworm, *Hyphantria cunea*. Pest Manag. Sci..

[30] Guo B., Wang Y.M., Zhang J., Ding W.B., He H.L., Gao Q., Gao H.S., Li Y.Z., Qiu L. (2025). Silencing the serine/threonine kinase Akt gene disrupts reproductive physiology in *Sogatella furcifera* and confers RNAi-mediated insect resistance in rice. Pest Manag. Sci..

[31] Qiu X.Y., Huang W.Q., Yue W.B., Li D.Y., Zhi J.R. (2024). Response of the serine/threonine kinase AKT and phosphoinositide-dependent kinase PDK in *Frankliniella occidentalis* (Thysanoptera: Thripidae) to three kinds of foods and their regulation of reproductive function. Insect Mol. Biol..

[32] Hu K., Jin R., Liu J.Q., Zhu J., Dai W., Wang Y., Li Y., Liu F. (2024). Functional characterization of the InR/PI3K/AKT signaling pathway in female reproduction of the predatory bug *Cyrtorhinus lividipennis* (Hemiptera: Miridae). J. Econ. Entomol..

[33] Nässel D.R., Broeck J.V. (2016). Insulin/igf signaling in *Drosophila* and other insects: Factors that regulate production, release and post-release action of the insulin-like peptides. Cell. Mol. Life Sci..

[34] Ghosh S., Leng W.H., Michaela W.B., Barrera-Velazquez M., Leopold P., Eaton S. (2022). Local insulin reservoir in *Drosophila* alpha cell homologs ensures developmental progression under nutrient shortage. Curr. Biol..

[35] Battersby A., Csiszar A., Leptin M., Wilson R. (2003). Isolation of proteins that interact with the signal transduction molecule dof and identification of a functional domain conserved between dof and vertebrate BCAP. J. Mol. Biol..

[36] Xue H., Huang X.X., Chang G.F., Ma W.H., Hull J.J., Chen L.Z. (2022). Reproductive capacity in *Adelphocoris suturalis* (Hemiptera: Miridae) is regulated by the insulin signaling pathway. Pestic. Biochem. Physiol..

[37] Xu K.K., Yan Y., Yan S.Y., Xia P.L., Yang W.J., Li C., Yang H. (2021). Disruption of the serine/threonine kinase *Akt* gene affects ovarian development and fecundity in the cigarette beetle, *Lasioderma serricorne*. Front. Physiol..

[38] Fujinaga D., Shiomi K., Yagi Y., Kataoka H., Mizoguchi A. (2019). An insulin-like growth factor-like peptide promotes ovarian development in the silkmoth *Bombyx mori*. Sci. Rep..

[39] Al Baki M., Jung J.K., Kim Y. (2021). Physiological alterations in deletion mutants of two insulin-like peptides encoded in *Maruca vitrata* using CRISPR/Cas9. Front. Physiol..

[40] Feng J.Y., Du J., Li S.W., Chen X.X. (2024). Akt regulates the fertility of *Coridius chinensis* by insulin signaling pathway. Sci. Rep..

[41] Roy S., Saha T.T., Zou Z., Raikhel A.S. (2018). Regulatory pathways controlling female insect reproduction. Annu. Rev. Entomol..

[42] Hansen I.A., Attardo G.M., Roy S.G., Raikhel A.S. (2005). Target of rapamycin-dependent activation of S6 kinase is a central step in the transduction of nutritional signals during egg development in a mosquito. J. Biol. Chem..

[43] Roy S.G., Raikhel A.S. (2001). The small GTPase Rheb is a key component linking amino acid signaling and TOR in the nutritional pathway that controls mosquito egg development. Insect Biochem. Mol. Biol..

[44] Lu K., Chen X., Liu W.T., Zhou Q. (2016). TOR Pathway-mediated juvenile hormone synthesis regulates nutrient-dependent female reproduction in *Nilaparvata lugens* (Stål). Int. J. Mol. Sci..

[45] Rauschenbach I.Y., Karpova E.K., Adonyeva N.V., Andreenkova O.V., Faddeeva N.V., Burdina E.V., Alekseev A.A., Menshanov P.N., Gruntenko N.E. (2014). Disruption of insulin signalling affects the neuroendocrine stress reaction in *Drosophila* females. J. Exp. Biol..

[46] Pan X., Pei Y.F., Zhang C.C., Huang Y.L., Chen L., Wei L.Q., Li C.R., Dong X.L., Chen X. (2022). Effect of insulin receptor on juvenile hormone signal and fecundity in *Spodoptera litura* (F.). Insects.

[47] Fu K.Y., Zhu T.T., Guo W.C., Ahmat T., Li G.Q. (2016). Knockdown of a putative insulin-like peptide gene Ldilp2 in *Leptinotarsa decemlineata* by RNA interference impairs pupation and adult emergence. Genes.

[48] Bento F.M.M., Marques R.N., Campana F.B., Demétrio C.G.B., Leandro R.A., Parra J.R.P., Figueira A. (2020). Gene silencing by RNAi via oral delivery of dsRNA by bacteria in the South American tomato pinworm, *Tuta absoluta*. Pest Manag. Sci..

[49] Zhu K.Y., Palli S.R. (2020). Mechanisms, applications, and challenges of insect RNAi. Annu. Rev. Entomol..

[50] Mahanta D.K., Komal J., Bhoi T.K., Samal I., Dash S., Jangra S. (2025). RNA interference (RNAi) for insect pest management: Understanding mechanisms, strategies, challenges and future prospects. Biol. Futur..

