# Supplementary material for: TaPIK3AP Regulates Female Reproduction in Tuta absoluta Through Juvenile Hormone-, Vitellogenin-, and TOR-Related Signaling
Source: Insects. 2026 Jul 10;17(7):711. doi: 10.3390/insects17070711 (PMC13410082; doi:10.3390/insects17070711)
Supplement: Supplementary file 1 [file insects-17-00711-s001.zip › insects-4399954-supplementary.pdf]

Table S1. Primer sequences used in this study.

Application	Primer name	Primer sequence (5′ to 3′)
Full-length cloning	*TaPIK3AP*-F	ATGGGGCCGCAGTTCGACTA
	TaPIK3AP-R	CTATGTATGCGTAGCAGAT
qPCR analysis	q*TaPIK3AP*-F	ATTCCAGAGTGACGCCAGTA
	q*TaPIK3AP*-R	TCGTAGCCATGTGAGGTGA
	q*TaEF1α*-F	CCTGGGCACAGAGATTTCAT
	q*TaEF1α*-R	GATCAGCTGCTTGACACCAA
	q*TaVg*-F	TGGTACGTGGTTATGCAGGA
	q*TaVg*-R	TACTTCGACACTGGGGGTTC
	q*TaVgR*-F	GCAAACCTGAGAGCCACTTC
	q*TaVgR*-R	CAGCGGCTGTCATTTCTACA
	q*TaJHAMT*-F	AGACCACAGGATCCAGTTCG
	q*TaJHAMT*-R	ATATCAGCAGACACTCCCCG
	q*TaKr-h1*-F	TGCGGCATTATAGGACCCAT
	q*TaKr-h1*-R	GAGCGTGACGATGGAGTTTC
	qTamTOR-F	CAGCCAAACAGTCAACTTGCA
	qTamTOR-R	TGAAGCTCTCACACAAATATCCTCT
	qTaRAPTOR-F	GAGTAGACCCGCCAGATGTG
	qTaRAPTOR-R	ACGCTCATCTTTGGCATTGC
	qTaRheb-F	TCTAGCAAAAGTTTTCAAATCGTACA
	qTaRheb-R	ACAGACTCATTTTTCTTTGCACT
	qTaTSC1-F	CGAGCCGTTACCGCTGTTA
	qTaTSC1-R	ACCGCAAAGTCTCGACCAG
	qTaTSC2-F	CTTCCCCGAATGCCTCAGAG
	qTaTSC2-R	CATGGGGCTGGCGATATTCT
	qTaAMPK-F	GGACGGAGAGTTTTTGAGGACAT
	qTaAMPK-R	GGGAAGATGCCGGATTTGATCT
	qTaS6k-F	CACACACTAAGGCTGAAAGGAA
	qTaS6k-R	GCTGTGTAAATGCTCCAAGGC
	qTa4EBP-F	AGACAGTCCCCGATCTCACA
	qTa4EBP-R	GTTTCCTGGCTCTCGTCGAA
	qTaAkt-F	AGCTGTTCTTCCACCTCTCC
	qTaAkt-R	GGCCGTACGTTATGTTCACC
dsRNA synthesis	ds*GFP*-F	TAATACGACTCACTATAGGGTACAAGACGCGTGCTGAAGT
	ds*GFP*-R	TAATACGACTCACTATAGGGCAATGTTGTGGCGAATTTTG
	ds*TaPIK3AP*-F	TAATACGACTCACTATAGGGAGACCCCTCAACGGCTGC
	ds*TaPIK3AP*-R	TAATACGACTCACTATAGGGCGCCGTCCATGTCGTCGC

The underlined part indicates the T7 promoter sequence; F: Forward primer; R: Reverse primer.
